# Formulation and characterisation of drug-loaded antibubbles for image-guided and ultrasound-triggered drug delivery

**DOI:** 10.1016/j.ultsonch.2022.105986

**Published:** 2022-03-23

**Authors:** Spiros Kotopoulis, Christina Lam, Ragnhild Haugse, Sofie Snipstad, Elisa Murvold, Tæraneh Jouleh, Sigrid Berg, Rune Hansen, Mihaela Popa, Emmet Mc Cormack, Odd Helge Gilja, Albert Poortinga

**Affiliations:** a Department of Clinical Medicine, University of Bergen, Bergen, Norway; b National Centre for Ultrasound in Gastroenterology, Haukeland University Hospital, Bergen, Norway; c Department of Physics, Norwegian University of Science and Technology, Trondheim, Norway; d Neoety AS, Kløfta, Norway; e Department of Clinical Science, University of Bergen, Bergen, Norway; f Department of Quality and Development, Hospital Pharmacies Enterprise in Western Norway, Bergen, Norway; g Department of Biotechnology and Nanomedicine, SINTEF Industry, Trondheim, Norway; h Cancer Clinic, St. Olav’s Hospital, Trondheim, Norway; i Department of Circulation and Medical Imaging, Norwegian University of Science and Technology, Trondheim, Norway; j Department of Health Research, SINTEF Digital, Trondheim, Norway; k KinN Therapeutics, Bergen, Norway; l CCBIO, Department of Clinical Science, University of Bergen, Norway; m Polymer Technology, Eindhoven University of Technology, Eindhoven, the Netherlands

**Keywords:** Ultrasound, Microbubbles, Antibubble, Targeted drug delivery, Sonoporation

## Abstract

The aim of this study was to develop high load-capacity antibubbles that can be visualized using diagnostic ultrasound and the encapsulated drug can be released and delivered using clinically translatable ultrasound.

The antibubbles were developed by optimising a silica nanoparticle stabilised double emulsion template.

We produced an emulsion with a mean size diameter of 4.23 ± 1.63 µm where 38.9 ± 3.1% of the droplets contained a one or more cores. Following conversion to antibubbles, the mean size decreased to 2.96 ± 1.94 µm where 99% of antibubbles were <10 µm. The antibubbles had a peak attenuation of 4.8 dB/cm at 3.0 MHz at a concentration of 200 × 10^3^ particles/mL and showed distinct attenuation spikes at frequencies between 5.5 and 13.5 MHz. No increase in subharmonic response was observed for the antibubbles in contrast to SonoVue®. High-speed imaging revealed that antibubbles can release their cores at MIs of 0.6. *In vivo* imaging indicated that the antibubbles have a long half-life of 68.49 s *vs.* 40.02 s for SonoVue®. The antibubbles could be visualised using diagnostic ultrasound and could be disrupted at MIs of ≥0.6. The *in vitro* drug delivery results showed that antibubbles can significantly improve drug delivery (p < 0.0001) and deliver the drug within the antibubbles. In conclusion antibubbles are a viable concept for ultrasound guided drug delivery.

## Highlights

• Antibubble formulation was developed with a mean diameter of 2.96 µm and 99% of antibubbles were <10 µm in diameter.
• The antibubbles mean encapsulation volume was 21%
• Antibubbles showed similar acoustic attenuation to SonoVue with numerous harmonic peaks.
• Antibubbles could be destroyed at MIs as low as 0.6.

## 1 Introduction

**Fig. 1:**
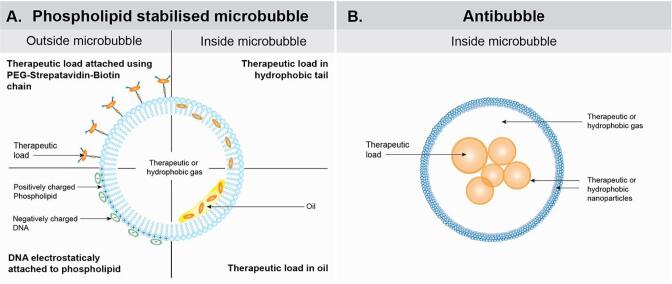
Schematic depicting different ways of loading drugs in phospholipid stabilised microbubbles (Panel A) compared to antibubbles (Panel B).

Microbubbles have a long history as ultrasound contrast agents [1] and the option of loading microbubbles with drugs with an aim of using them for ultrasound-triggered drug delivery has been widely studied [2], [3], [4], [5]. However, this approach has not reached clinical application yet. Different ways exist in which microbubbles can be loaded with drugs, (*c.f.*, Fig. 1**A**). Most frequently the drug resides at the outside surface of the microbubbles, either directly coupled to the surface, or, loaded into liposomes or nanoparticles coupled into the surface [6], [7], [8], [9]. Alternatively, the drug is loaded into the shell of the microbubbles, either inside the shell, which often consists of a layer of phospholipids, or dissolved in an additional oil layer present within the shell [10], [11], [12]. These approaches suffer from several drawbacks, such as the relatively low stability of the microbubbles in circulation, low drug loading because of little space within the thin microbubble shell, unstable drug release as the drugs are often difficult to release from the microbubble shell *in vivo*, and in many cases only hydrophobic drugs can be incorporated efficiently [13]. Moreover, loading a microbubble shell with drugs can negatively affect the resonance behavior of the bubble [14] and can increase the pressure-threshold for the onset of microbubble oscillation [15]. Furthermore, inertial cavitation is in general needed to release the loaded drugs, however this holds a risk of damaging healthy tissue. An alternative strategy can be to load the drugs inside one or more cores within the microbubbles. Bubbles containing a droplet within their volume were first described in 1932 and have been referred to as inverse bubbles or antibubbles because they are the inverse of conventional soap bubbles [16]. These bubbles are poorly stabilized by surfactants and for about 80 years antibubbles therefore only existed as very short-lived structures, generally with in vial/*in vitro* lifetimes of only a few minutes, that were practically useless [17]. In 2007, when observing transient antibubbles with a size of a few microns and a lifetime in the order of microseconds, Postema stated that antibubbles could be an ideal system for ultrasound-triggered drug delivery if only they could be produced consistently and with sufficient lifetime [14]. The antibubble configuration provides (Fig. 1**B**) ample space for drugs to be incorporated, the drugs can be hydrophilic as well as hydrophobic, they are shielded from the environment by the gas shell, the gas shell allows the bubble to be acoustically active and in principle stable cavitation should be sufficient to release the encapsulated drugs [18]. In 2011, Poortinga for the first time produced antibubbles with a lifetime of at least tens of hours (and a size in the mm range) using hydrophobized silica nanoparticles to stabilize the interfaces of the antibubbles, so-called Pickering stabilization [19]. This was followed by the publication of a method to produce stable antibubbles with a size of ten to several tens of microns [20], [21]. Since then, the potential of antibubbles for drug delivery applications has been more and more recognized [18], [22], [23]. Also, it has been shown for antibubbles in which the cores are solid instead of liquid, that antibubbles give a clear acoustic response in a medical ultrasound field. Several steps however still need to be taken before antibubbles can be applied for ultrasound-triggered drug delivery. For example, the size of the antibubbles needs to be small enough (<10 µm) to avoid blocking of blood capillaries after intravenous injection [24], the encapsulated drug needs to be released at medically relevant ultrasound intensities and the antibubbles should not give systemic toxic effects *in vivo*. We describe the process of optimizing and producing antibubbles with diameters < 10 µm, evaluate their response to ultrasound using acoustic spectroscopy and ultrasound imaging at various frequencies *in vitro* and *in vivo,* and briefly evaluate their capacity to enhance drug delivery *in vitro* using a model drug.

## 2 Materials and methods

### 2.1 Materials

Unless otherwise stated, all chemicals and consumables were purchased from Merck KGaA, Darmstadt, Germany. Maltodextrin MD19 was purchased from Roquette, Frères, Lestrem, France; perflubutane (C_4_F_10_) (PFC; F2 Chemicals, Preston, UK). The different types of amorphous hydrophobized silica nanoparticles used to stabilize the interfaces of the antibubbles are given in Table 1. These nanoparticles were chosen based on their carbon content, indicative of their hydrophobicity, primary particle size (5–50 nm), and their use in previous antibubble work [21]. The Brunauer, Emmett and Teller (BET) surface area can be used to estimate the particle size [25] allowing an indirect comparison between different particle shapes, *i.e.*, a larger BET surface area correlates to a smaller particle size.

**Table 1:** The different types of amorphous hydrophobized silica nanoparticles used, with their respective carbon content, surface area, and supplier of either Wacker (Munich, Germany), or Evonik (Essen, Germany).

Type	Carbon content (%)	BET surface area (m^2^/g)	Calculated Particle diameter (nm)	Supplier
HDK H18	4.0 – 5.2	170 – 230	12 – 16	Wacker
HDK H17	3.5 – 5.5	130 – 170	16 – 21	Wacker
Aerosil R812S	3.0 – 4.0	195 – 245	12 – 15	Evonik
Aerosil R972Ph	0.6 – 1.2	90 – 130	23 – 33	Evonik
HDK H15	0.2	130 – 170	16 – 21	Wacker

### 2.2 Antibubble production and optimization

**Fig. 2:**
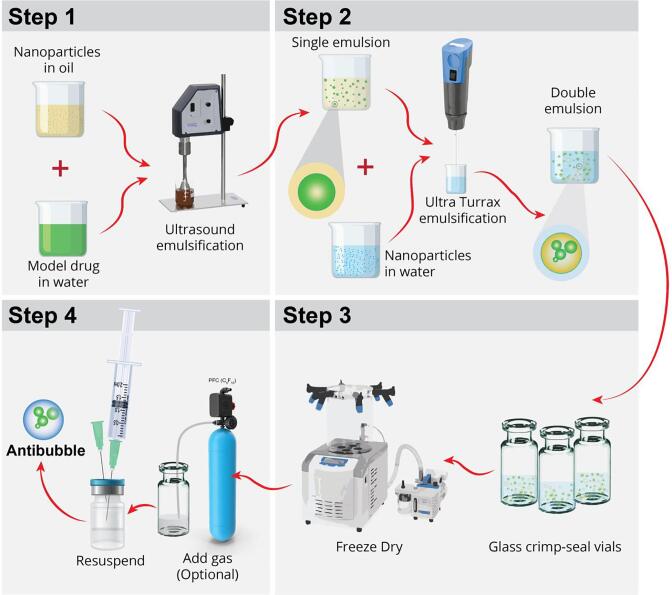
Schematic illustration of the antibubbles production process. ***Step 1***: Disperse an aqueous phase containing the model drug in an oil phase containing silica nanoparticles using an ultrasound probe to produce a water-in-oil emulsion. ***Step 2:*** This water-in-oil emulsion is then dispersed using a high-performance dispersing instrument (ultraturrax) in an aqueous phase containing silica nanoparticles to produce a water-in-oil-in-water emulsion. ***Step 3:*** Freeze-dry the emulsion and lyophilize it. Lyophilization removes the volatile oil phase. ***Step 4:*** Dissolving the lyophilized structure in water leads to formation of a suspension of microbubbles containing one or more cores, *i.e.,* the formation of an antibubble suspension.

The method used to produce antibubbles is shown schematically in Fig. 2 whilst the emulsification conditions and general composition of the different phases are given in Table 2. The production starts with dispersing an aqueous phase (referred to as the ‘Inner phase’) in an oil phase containing silica nanoparticles (‘Middle phase’) using a 24 kHz ultrasonic emulsifier (UP 400St, Hielscher Ultrasonics, Teltow, Germany) to produce a water-in-oil emulsion (‘W/O emulsion’). This W/O emulsion is then dispersed using a high-performance dispersing instrument (T 10 basic ULTRATURRAX®, IKA-Werke GmbH & Co. KG, Staufen, Germany) in an aqueous phase containing silica nanoparticles (‘Outer phase’) to produce a water-in-oil-in-water emulsion (‘W/O/W emulsion’). Different types and concentrations (2–6%) of silica nanoparticles were tested to determine an optimized formulation (Table 1). The W/O/W emulsion serves as a template for the production of antibubbles. This is done by rapidly freezing the W/O/W emulsion in liquid nitrogen followed by lyophilizing it. Lyophilization serves to remove the volatile oil in the middle phase. The inner and outer water phases contain a carbohydrate to assure that the structure remains intact during removal of the volatile oil. Dissolving the lyophilized structure in water leads to the formation of a suspension of microbubbles containing one or more cores, *i.e.,* the formation of an antibubble suspension. The fluorescent dye calcein was added to the inner phase to make it easier to detect the presence of cores inside the antibubbles. Calcein stock solution was prepared by dissolving in 1 M NaOH to a concentration of 50 mg/mL, according to manufacturer’s instructions. The stock-solution was added to the Inner Phase (20 µL stock-solution per mL Inner Phase) to result in a final concentration of 1 mg calcein per mL and mixed for up to 20 min on a magnet stirrer in the dark to prevent photobleaching. The calcein concentration was defined after optimization for fluorescent imaging conditions. Calcein has previously been used as a model drug to evaluate the efficacy of sonoporation [26], [27].

**Table 2:** General composition and processing conditions to produce W/O/W emulsions. The quantity units are described using the measurement method used, *i.e.*, weight or volume to minimise the impact of the different densities of water, silica, and oil.

Single phases				
Phase	Composition	Quantity	Container	Dispersing
Inner water phase (I)	Water 10% w/v MD19 Maltodextrin 0.9% w/v NaCl 1 mg/mL w/v calcein	5 g	20 mL glass beaker	Magnetic stirrer
Middle oil phase (II)	Cyclohexane 2–6% w/v hydrophobized silica	20 g	50 mL glass beaker	Ultrasonic homogenisation 3000 Ws at 50% amplitude
Outer water phase (III)	Water 10% w/v MD19 Maltodextrin 0.9% w/v NaCl 0.5–4% w/v hydrophobized silica	40 g	100 mL glass beaker	Ultrasonic homogenisation 10 000 Ws × 3 at 100% amplitude

Emulsions				
W/O	I → II	5 g → 20 g	250 mL Glass media bottle	Ultrasonic homogenisation 3000 Ws at 50% amplitude
W/O/W	W/O → III	3 mL → 12 mL	20 mL glass beaker	Rotary dispersion 7900–29 900 RPM 1–10 min

Nanoparticle stabilised microbubbles were produced using the same procedure but replacing the W/O phase with cyclohexane only.

### 2.3 Lyophilization and resuspension

Crimp-head glass vials (2 mL) were filled with 700 μL of W/O/W emulsion, flash frozen in liquid nitrogen and loaded onto a pre-cooled freeze-dryer (CHRIST Alpha 2–4 LD plus). The freeze dryer was set to a pressure of 0.001 mbar and a condenser temperature of −85 °C. The drying lasted 18–40 h depending on the batch size. At the end of the process, the vacuum pump was switched off while opening the vent to slowly allow air to enter the drying chamber and let the pressure increase to atmospheric pressure over a period of approximately 2 min. Optionally, the crimp-head vials were immediately filled with PFC gas. The PFC gas was filled by placing a 4 mm diameter tube with a 1 mL pipette tip connected to PFC gas canister into the bottom of the vial. The PFC gas could be visualized flowing into the vial due to optical diffraction. Once the PFC gas was flowing out of the vial the vials were sealed with a rubber stopper and an aluminum cap using a vial crimp. The vials were left for at least 15 min to allow the gas to diffuse into the bubbles before use. The antibubble formulations subsequently referred to as “air” were sealed as they exit the freeze dryer without adding PFC.

### 2.4 Microscopic analysis

Prior to microscopic characterization of the antibubbles, the lyophilized cake was resuspended: an 18G venting needle was inserted in the vials containing the lyophilizate and 2 mL maltodextrin 10%/ NaCl 0.9% (sugar solution) was injected followed by gently swirling the vial for 45–60 s until the lyophilizate was dissolved. The obtained antibubble suspension was diluted 20 times in the maltodextrin/NaCl solution to acquire images with predominantly single, non-overlapping antibubbles. A Nikon Eclipse E200 optical microscope with a Nikon 40 x/0.60NA or 10 x/0.25NA air objective was used. Fluorescence microscopy was performed using excitation with a blue light (CoolLed pE-300ultra) source in combination with a long pass filter cube (480/30 nm excitation and detection at >515 nm). Image analysis using MIPAR 2.0 (Worthington, OH, USA) was performed to obtain the size distribution of the antibubbles from the brightfield image and of the cores inside the antibubbles from fluorescence image. From the size distribution the number-averaged droplet/bubble size (μ) and d_10_ and d_90_ were calculated, meaning that 10% or 90% of the droplets/bubbles had a diameter smaller than d_10_ or d_90_, respectively. The polydispersity index (PDI) was calculated using PDI = (σ/μ)^2^ with σ being the standard deviation of the size distribution.

### 2.5 Scanning electron microscopy (SEM)

The lyophilized W/O/W emulsions were removed from the vial and broken up into small mm sized pieces. A piece of aluminum was used as the imaging substrate. A thin layer of conductive silver paint (Agar Scientific Ltd, Essex, United Kingdom) was painted onto the aluminum substrate and the millimeter sized pieces were immediately placed on the paint. The samples were placed in an oven (Supplier) heated to 60 °C for 5 min to dry the paint and glue the sample to the substrate. An 8 nm layer of chrome was sputter-coated onto the sample to make it conductive using a JEOL JFC-2300HR high resolution fine sputter-coater (JEOL Ltd, Tokyo, Japan). The samples were imaged using JEOL JSM-7400F scanning electron microscope (JEOL Ltd).

### 2.6 *In vitro* stability of the antibubbles

*In vitro* stability was evaluated by measuring the change in size and concentration of the antibubbles in cell culture medium (Dulbecco's Modified Eagle's medium (DMEM)) or in sugar solution. The cell culture solution contains proteins, salts, glucose, electrolytes, vitamins, and gases that better mimic physiological conditions. Stability of the antibubbles was evaluated for antibubbles filled with air or PFC. First, lyophilizate was resuspended by adding 2 mL of sugar solution to the vial. The resuspension medium was first incubated at 37 °C with 5% CO_2_ for 20 min in Forma Steri-cycle (Thermo Fischer Scientific, Waltham, MA, USA). Following resuspension of the lyophilizate, 20 μL of the samples was diluted in 200 μL of their respective media types in a 1.5 mL microcentrifuge tube. A second incubation step (37 °C, 5% CO_2_, 10 min) was performed following the dilution. Images of the samples were captured using optical microscopy as previously described.

### 2.7 *In vitro* acoustic response to ultrasound

#### 2.7.1 Attenuation and cavitation

The resonance frequency of the antibubbles was determined by measuring the acoustic attenuation as a function of frequency using the experimental configuration previously used to characterise SonoVue, Sonazoid and Optison [28]. SonoVue (Bracco S.p.A,Milan, Italy) was used as a reference. The bulk resonance frequency was assumed to be the frequency with the highest attenuation. A 23-mm Ø polyvinylidene fluoride (PVDF) transducer, with a focal depth of 49.5 mm (Precision Acoustics Ltd, Dorchester, UK) was connected to a pulser/receiver (5072PR, Olympus Scientific Solutions, Waltham, MA, USA) connected to a 200 MHz oscilloscope (DSOX3024A, Keysight Technologies, Santa Rosa, CA, USA). An 80 mL sample chamber was 3D printed (Supplier) with 32 mm Ø windows for unobstructed acoustic propagation. The acoustic windows were sealed using 23 μm-thick mylar sheets glued on to the sample chamber. The sample chamber was positioned so that the acoustic focus of the transducer coincided with the middle of the chamber. A 2-cm thick stainless-steel block was used as the reflector and placed 8 cm from the transducer face. The pulser/receiver was set at a pulse repetition frequency (PRF) of 100 Hz, an energy level of 2, damping of 50 Ω and a gain of 40 dB. In this configuration, the acoustic output from the PVDF transducer resulted in a peak-negative pressure of 0.56 MPa at 10.38 MHz centre frequency, *i.e.*, and MI of 0.17. Adjusting the energy level and damping primary affected the pulse bandwidth and had minimal effect on the acoustic pressure, hence only this pressure was used to evaluate attenuation. An energy level of two was chosen as this resulted in the widest pulse bandwidth in the acoustic range the antibubbles were expected to respond. The acoustic output was calibrated in a 3-axis water tank using a 200-µm diameter needle hydrophone kit (NH-0200, Precision Acoustics,). The sample chamber was filled with saline supplemented with 5% bovine serum albumin (Merck KGaA, Darmstadt, Germany) which was warmed to 44 °C for 24 h and reduced to 37 °C for 4 h prior to use to attempt a match to typical blood gas saturation. Twenty baseline waveforms (without microbubbles) were recorded for background subtraction. Bubbles removed from the vial were added to the sample chamber and gently agitated using a 1-mL pipette six times before each measurement. A range of concentrations was evaluated. For each concentration 15 recordings (1 s apart) were captured during gentle agitation with a 1-mL pipette placed outside the sound field at the edge of the container. A graphical rendering of the experimental setup is shown in Supplemental Fig. 1A.

To evaluate the inertial cavitation range of the SonoVue and the antibubbles, the same experimental setup was used with some minor modifications (*c.f.*, Supplemental Fig. 1**B**). A bespoke 65-mm Ø, focused, lead zirconate titanate (PZT) ultrasound transducer with a focal depth of 75 mm (Precision Acoustics Ltd) was added to the setup and the transducers were arranged perpendicularly to each other. The acoustic output was calibrated in a 3-axis water tank using a 200-µm diameter needle hydrophone kit (NH-0200, Precision Acoustics). The acoustic foci were aligned by measuring the maximum reflected signal off a 1.5 mm spherical point reflector using the pulser/receiver (5072PR). The PZT transducer used a 3.28 MHz transmit frequency to improve spatial accuracy during alignment. For cavitation induction, the acoustic output of the PZT was configured to 1.08 MHz center transmit frequency with a 20-cycle pulse at a 100 Hz PRF. The PVDF transducer was connected to the pulser/receiver in the same configuration as for the attenuation measurements to act as a passive receiver of the acoustic cavitation emissions. For each MI evaluated, 15 samples were recorded.

The acoustic attenuation spectra were calculated in MATLAB (2021a) by obtaining the power spectrum (pspectrum function in MATLAB) for all individual waveforms, calculating the mean of the replicated measurements, and subtracting the averaged baseline from the averaged measurements with bubbles. The attenuation was converted to dB/cm by dividing the attenuation via the sample chamber thickness × 2; a total of 16 cm.

#### 2.7.2 *In vitro* ultrasound imaging

To determine if antibubbles could be imaged *in vivo* and to characterize their response to various acoustic amplitudes, the antibubbles were imaged in a water-perfused tissue-mimicking phantom (ATS model 524, CIRS Inc, VA, USA) with a GE Vivid E9 ultrasound scanner (GE Vingmed Ultrasound AS, Horten, Norway) and 9L probe (GE Vingmed Ultrasound AS) using nonlinear contrast imaging (pulse inversion). Approximately 100 μL of antibubble suspension was added to 2 L of deionized water, and a peristaltic pump controlled the flow speed through the phantom. The acoustic attenuation of the tissue mimicking material is given as 0.5 dB/cm/MHz and hence similar to what is found in soft tissue. The contrast enhanced images were recorded with transmit and receive frequencies of 3.6 MHz and 7.2 MHz, respectively, and mechanical indices (MI), (as given by the Vivid system) of 0.05 to 1.2.

#### 2.7.3 High-speed imaging

The acoustic response of antibubbles resuspended in DMEM was visualized using high-speed optical imaging. This experimental setup combines a Fastcam Mini AX100 (Photron, Tokyo, Japan) high speed camera with a 60X/1.00 W water objective (Olympus LumPLANFL N 60x/1.00 W) in an upright microscope configuration. The light is provided by a liquid light guide coupled to a 175 W xenon (ASB-XE-175, Rapitech Enterprise Co., Ltd., Taiwan) light source. Ultrasound was generated by a single element focused ultrasound transducer with a center frequency of 1.08 MHz (Precision Acoustics), *i.e.,* the same transducer used for the cavitation measurements. The pulse repetition rate was set at 100 μs. The ultrasound pressure was varied up to a maximum of 0.89 MPa peak-negative corresponding to an MI of 0.86. The ultrasound transducer was calibrated in 3-axis in an open water bath using 200–µm PVDF needle hydrophone (Precision Acoustics Ltd, Dorchester, UK). A rendering of the experimental setup can be seen in Supplemental Video 1. BxPC-3 cells (CRL-1687™, ATCC, Manassas, VI, USA) were cultured in a 2D hypoxic bioreactor (PetakaG3 LOT, Celartia, Columbus, OH, USA) as previously described [27]. Antibubbles were injected via the injection port after diluting 100 µL of resuspended antibubbles in 1 mL of sugar solution resulting in a total dilution of 1:250. The antibubbles were allowed to float for 30 – 60 s with the cell covered surface positioned highest to allow the antibubbles to contact the cells. The PetakaG3 LOT was subsequently moved until antibubbles could be seen in the FOV in contact with the cells.

### 2.8 *In vivo* stability of the antibubbles and response to ultrasound

#### 2.8.1 Rat imaging

Lyophilized antibubbles were resuspended in saline solution. A male Sprague Dawley rat (597 g) was anesthetized by isoflurane in oxygen (5% during induction and 2% during maintenance), and the body temperature was maintained by a heating blanket during the procedure. The rat was euthanized by an intravenous injection of pentobarbital (100 mL/kg) when the imaging procedure was finished. The abdomen was shaved, and remaining hair removed using depilatory cream (Veet), and a 24 G catheter (Becton Dickinson & Company) was placed in the tail vein. A clinical ultrasound system GE Vivid E9 combined with an 11L ultrasound probe (GE Healthcare) was used to image the mesenteric arteries. The imaging mode was switched to pulsed wave (PW) Doppler with the region of interest (ROI) cursor placed on the vessel of interest. An example photograph of the setup can be seen in Supplemental Fig. 2**.** A bolus of 150 μL of antibubbles was injected via the tail vein, and the PW spectrum was stored for the following 160 s, during which time the transducer was immobilized. Post-processing of the recorded data was done using MATLAB, where the linear signal intensity of all the pixels in the PW Doppler spectrum from each recorded second was summed to give one datapoint on the time-intensity curve. This data was used to determine the *in vivo* half-life of the antibubbles.

#### 2.8.2 Mouse imaging

Antibubbles were resuspended in 2 mL sugar solution. Male mice (n = 3, 20–25 g, Gades Institute, University of Bergen; originally a generous gift of Prof. Leonard D. Shultz, Jackson Laboratories, Bar Harbour, ME, USA) were housed in individually ventilated cages inn specific pathogen free conditions at 22–23 °C and 50–60% relative humidity with free access to food and water. All animal procedures were approved by the Norwegian Animal Research Authorities (Norwegian Food Safety Authority, application No.: 16/159013, 02.01.2017).

Prior to imaging, mice were anesthetized by isoflurane in oxygen (3% during induction and 1% during maintenance), and the body temperature was maintained by an isostatic heater at 37 °C during the procedure. A 50 µL bolus of antibubbles was injected into the lateral tail vein via a 30G insulin syringe (B.Braun, Melsungen, Germany) over a 5 s period. The liver was imaged using either an MS250 ultrasound transducer connected to a Vevo 2100 (Fujifilm Visualsonics, Toronto, Canada) in standard abdominal preset with contrast mode enabled or using a GE Logiq E9 combined with a 9L ultrasound transducer in the vascular preset with contrast mode enabled. Post imaging, mice were observed for general appearance and pain in accordance with the NC3Rs guidelines [29] for 14 days.

#### 2.8.3 *In vitro* drug delivery

To evaluate the potential benefit and feasibility of delivering a drug encapsulated within the antibubbles to cells an *in vitro* pilot study was performed comparing loaded antibubbles *vs*. non-loaded microbubbles. The non-loaded microbubbles were produced using the same technique as the antibubbles but did not include the inner phase, *i.e.*, the antibubble core. This was done to minimize the difference between the two formulations in case there was any endocytosis or other effects of the free silica and to separate the impact of the non-loaded antibubbles within the antibubble formulation. The identical experimental configuration as in our previous work was used [27]. In summary, BxPC-3 cell (ATCC, Manassas, VA, USA) were cultured in a PetakaG3 LOT with 5 × 10^6^ particles/mL antibubbles or microbubbles. The entire cell culture surface was exposed to 2.00 MHz ultrasound at an MI of 0.39, with 32 packets of 160 cycles at 22 Hz, resulting in a duty cycle of 3.6%, an I_SPTA_ of 358 mW/cm^2^, and I_SPPA_ of 10 W/cm^2^, *i.e.*, an acoustic parameter space within the clinical diagnostic regime. The percentage of cells that had taken up calcein was then quantified using flow cytometry using the identical methods as previously described [27].

### 2.9 Statistical analysis

Statistical Analysis was performed in Prism (v 9.1.1, Graphpad Software LLC). In general data is presented as means ± SD unless otherwise stated. Significance was set at P ≤ 0.05. Comparisons were performed using a *t*-test or one way ANOVA unless otherwise stated. In all results presented ns > 0.05, * P ≤ 0.05, ** P ≤ 0.01, *** P ≤ 0.001, **** P ≤ 0.0001.

## 3 Results and discussion

### 3.1 Production and size characterization of antibubbles

**Fig. 3:**
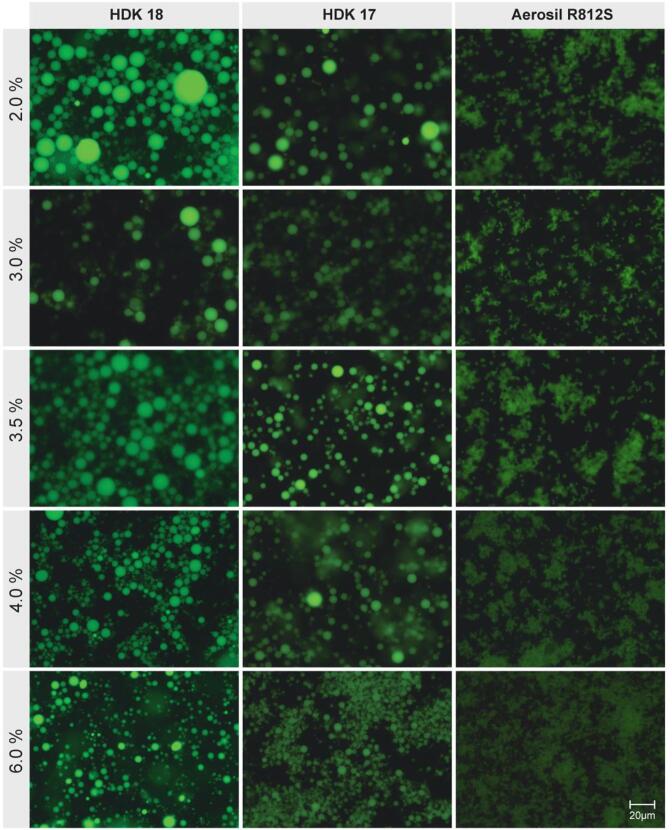
Fluorescence microscopy images of the W/O emulsions produced using a range of concentrations (2.0, 3.0, 3.5, 4.0 or 6.0%) of three different nanoparticles (HDK H18, HDK H17 and Aerosil R812S) are shown. Calcein is displayed in green and scale bar indicates 20 µm.

**Fig. 4:**
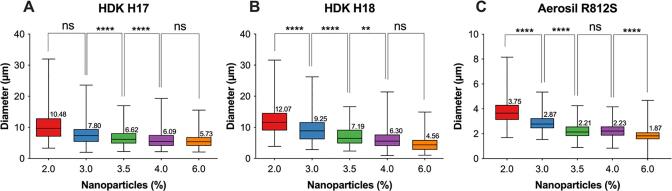
Box plot diagrams of mean diameter of the water droplets in the W/O emulsions when using the following silica nanoparticles (A) HDK H18, (B) HDK 17 (C) Aerosil R812S. The coloured boxes show maximum and minimum diameter for each concentration, the crossing black line through each box marks the mean diameter (μm) with the exact value specified above the box. *n* = 356 – 765 W/O droplets per nanoparticle type.

The first aim was to obtain small enough antibubbles to allow for intravenous injection. The influence of the type and concentration of the nanoparticles in the middle phase was studied. The formulation process as described in Table 2 was used. Only the three nanoparticle types with a higher carbon content, *i.e.*, more hydrophobic nanoparticles, were investigated. Fluorescence microscopy images of the W/O emulsions produced using a range of concentrations of three different types of nanoparticles are shown in Fig. 3**,** and the quantitative results of the average droplet size and the range of droplet sizes as a function of particle concentration for the different particle types, is shown in Fig. 4. For all types of nanoparticles, a higher particle concentration allows the production of a smaller droplet size. This was expected as a smaller droplet size means a larger interfacial area that needs to be covered with adsorbed nanoparticles. Whilst the use of HDK H18 and HDK H17 nanoparticles showed very similar results in terms of the droplet size they can stabilise, Aerosil R812S produced visibly smaller droplets, *i.e.*, the lowest concentration of Aerosil R812S produced 3.8 µm diameter droplets which was smaller than for the highest concertation of HDK H17 (5.7 µm) and HDK H18 (12.9 µm). A two-way ANOVA showed a significant impact of both the concentration (p < 0.0001) and nanoparticle type (p < 0.0001). To prepare a Pickering emulsion the nanoparticles must partially wet both the water phase and the oil phase [30], therefore hydrophobized silica was used. On these nanoparticles the hydrophilic silanol groups on the particle surface are reacted with organic groups [31]. The carbon content of the nanoparticles can be used as a measure of their hydrophobicity because a higher carbon content indicates a higher degree of surface modification. The HDK H18 and HDK H17 nanoparticles contain at most 20% of the silanol groups present at the surface of unmodified silica nanoparticles, while for Aerosil R812S the amount of silanol groups will be somewhat higher [30]. It has previously been observed that a minimal droplet size was obtained for hydrophobized silica nanoparticles with 50% of silanol groups remaining at the surface [30]. Hence we expected that in our case Aerosil R812S should give the smallest water particle size since of the three different particle types tested these particles have a percentage of silanol groups closest to 50%. However, when using Aerosil R812S nanoparticles more aggregated water droplets were observed than when using HDK H18 or HDK H17, possibly because their larger number of hydrophilic groups makes them less stable against aggregation in oil. From these results we concluded that a W/O emulsion stabilized by 5% or more HDK H17 nanoparticles was suitable to produce a W/O/W emulsion with small enough droplets to produce antibubbles with a size suitable for injection.

This optimized W/O emulsion was then used to optimize the second emulsification step to prepare a W/O/W emulsion. When preparing a W/O/W emulsion the nanoparticles used to stabilise the outer O/W interface need to be less hydrophobic than the nanoparticles used to stabilise the inner W/O phase. The formulation referred to as F1 (Table 3) was chosen, and an experimental design was executed to find the optimal homogenisation speed and duration. Supplemental Fig. 3 shows the average diameter of the W/O/W droplets as well as the absolute polydispersity and the relative polydispersity (relative to the average diameter) as a function of the turraxing speed and duration. A turraxing speed ≥ 5 (∼20 000 rpm) for 2–3 min gave a rather optimal W/O/W in terms of the average droplet size and the polydispersity. Longer turraxing increased the polydispersity. It can be assumed that the continuous break-up and coalescence of the droplets leads to the formation of aggregated nanoparticles that are less effective in stabilizing the emulsion. Therefore, while longer turraxing may produce more smaller droplets, it will also produce larger droplets leading to an increased polydispersity. A low polydispersity is important to ensure that the produced antibubbles have a similar resonance frequency such that they all respond similarly to the ultrasound and therewith a complete release of encapsulated active ingredient is obtained.

**Table 3:** Optimized W/O/W emulsion formulations.

Formulation	Inner phase	Middle phase	Outer phase
F1	10% maltodextrin 0.9% NaCl 1 mg/ml calcein	Cyclohexane 5% HDK H17	10% maltodextrin 0.9% NaCl 2% Aerosil R972Ph
F2	10% maltodextrin 0.9% NaCl 1 mg/ml calcein	Cyclohexane 5% HDK H17	10% maltodextrin 0.9% NaCl 2% HDK H15

A microscopic image of a W/O/W emulsions obtained under these processing conditions is show in Supplemental Fig. 4. The majority of the droplets were in the right size range and therefore this emulsion seemed a good template for the production of antibubbles with a size suitable for intravenous injection. It can also be seen that the emulsion contains some non-spherical objects. These objects could respond to ultrasound in a less predictable manner. The occurrence of these objects may be explained as follows. During turraxing emulsion droplets will repeatedly be elongated, compressed, broken-up and coalesced, *i.e.*, the interfacial area will constantly be changing. With every enlargement of the interfacial area, nanoparticles will be adsorbed. At the interfacial area adsorbed nanoparticles will be present at a high concentration, particularly when droplets elongate under shear they will adsorb nanoparticles and then relaxate back to a spherical shape. The nanoparticles, which are hydrophobized, tend to aggregate due to hydrophobic attraction forces acting between the parts of the nanoparticles that reside in the aqueous phase and hence the nanoparticles will tend to form a resilient shell of jammed nanoparticles around the droplets that may fixate droplets in a non-spherical shape. Such non-spherical nanoparticle-stabilized drops have been described previously [32]. Hence, we hypothesized that using less hydrophobic nanoparticles would lead to a more flexible shell and thus to the formation of less non-spherical droplets. Therefore, the Aerosil R972Ph nanoparticles present in the outer phase were replaced by HDK H15 nanoparticles, keeping the turraxing conditions the same.

**Fig. 5:**
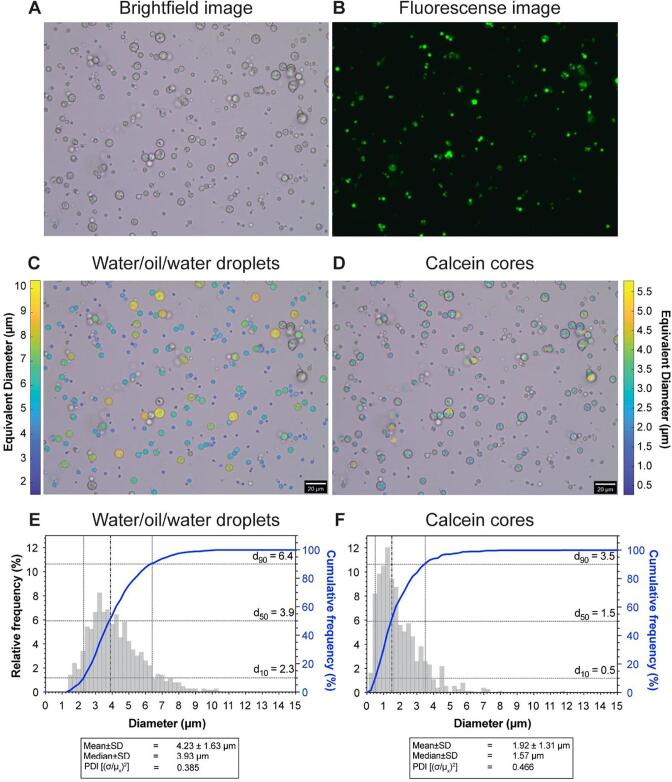
Microscopic images of the resulting emulsion of formulation F2 (HDK H15 nanoparticles in the outer phase). **Panel A** shows the brightfield image of the W/O/W emulsion whilst **Panel B** shows the fluorescence image of the W/O/W emulsion. **Panel C** shows the detected W/O/W droplets from the brightfield image. **Panel D** shows the same image as Panel C but the calcein cores have been detected using the fluorescence image. The colour of the detected droplets correlate the size of the particle. **Panel E** shows a size histogram of the detected W/O/W droplets which had a mean diameter of 4.23 µm with 99.7% of the droplets having a diameter < 10 µm.**Panel F** shows a size histogram of the detected calcein cores which had a mean diameter of 1.92 µm with 94.4% of the droplets having a diameter smaller than 4.23 µm. *n* = 3 vials; 6 – 12 images per vial, 991 total W/O/W droplets, 732 total calcein cores.

Microscopic images of the resulting emulsion (Fig. 5) shows that it is free of non-spherical objects (Fig. 5**A**) using formulation F2 (Table 3). To quantify the size of the outer droplets and evaluate the presence and size of the inner droplets, image analysis has been applied to the images (Fig. 5**C & D)**. The analysis shows that 38.9 ± 3.1% of the oil droplets were loaded, *i.e.*, contained inner droplets. This value is on the conservative side as there is a possibility that some fluorescent cores were not observed within the depth of field, or due to thresholding limits during image-post processing, some cores were not counted.

The size distribution of the W/O/W emulsion can be seen in Fig. 5**E & F**. The outer droplets have a mean diameter of 4.23 ± 1.63 µm with a d_10_ of 2.3 µm, a d_50_ of 3.9 µm and a d_90_ of 6.4 µm which corresponds to a polydispersity index of 0.385. A total of 99.7% of the oil droplets were smaller than 10 µm, indicating that the risk of blocking blood capillaries with the antibubbles will be acceptably small. The mean diameter of the cores was 1.92 ± 1.31 µm with a d_10_ of 0.5 µm, a d_50_ of 1.5 µm and a d_90_ of 3.5 µm, which corresponds to a polydispersity index of 0.466.

**Fig. 6:**
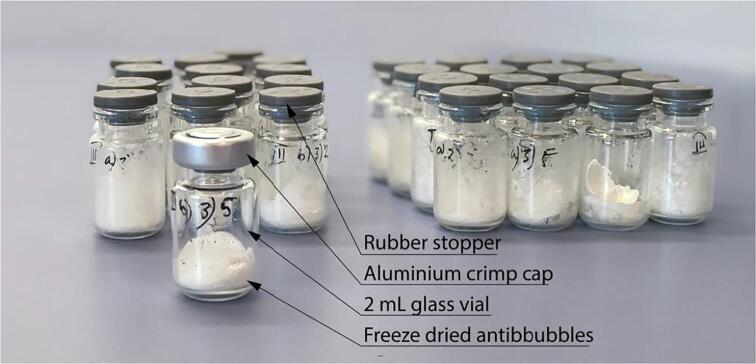
Photograph of a batch of antibubbles after being lyophilized. The cake can be seen to easily detach from the vial. The imperfect appearance with lyophilised product on the vial is due to the process of filling the vials by contacting the vial surface and motion prior to dipping in liquid nitrogen.

An example of laboratory scale antibubble batch is showing in Fig. 6. These were produced using formulation F2 (*c.f.,* Table 3) type antibubbles after lyophilisation. A traditional white “cake” is observed at the bottom of each vial.

**Fig. 7:**
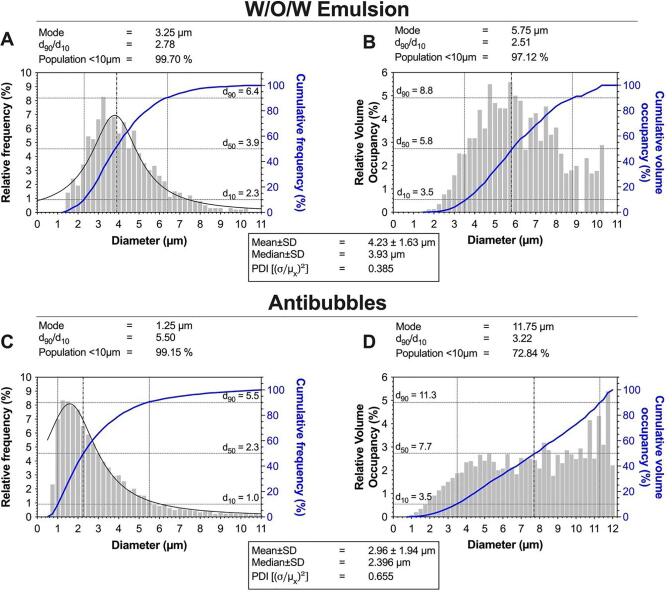
Size histograms of W/O/W emulsions and antibubbles following resuspension of formulation F2. **Panel A** and **Panel B** show the count and volume normalised size distribution of the W/O/W emulsion respectively. **Panel C** and **Panel D** show the count and volume normalised size distribution of the PFC antibubbles respectively. A reduction is size and increase in polydispersity is observed after the W/O/W emulsion becomes antibubbles. *n* = 3 vials; 5 – 26 images per vial, 991 total W/O/W droplets, 8892 total antibubbles.

The size distribution of W/O/W emulsion and subsequent antibubbles, presented as a count normalised and volume normalised distribution can be seen in Fig. 7. After converting the W/O/W into antibubbles, a reduction in mean diameter is observed; from 4.23 ± 1.63 µm to 2.94 ± 1.94 µm. In addition, an increase in polydispersity is also observed; from 0.385 to 0.655. This was previously observed with larger antibubbles [21] and is because the antibubbles will slightly swell and then show a tendency to shrink. This first swelling may to some extent ‘crack’ the particle shell surrounding the antibubbles and this will allow the antibubbles to dissolve or disproportionate. As a result, the smaller bubbles may shrink and the larger bubbles will grow, which may explain the increase in polydispersity.

Whilst the polydispersity of the antibubbles was 3.6 × higher than the commercially available ultrasound contrast bubbles SonoVue® (PDI = 0.18) [33], 99.15% of the antibubbles were still < 10 µm, indicating that the antibubbles could still be considered safe to inject intravascularly without blocking capillaries. These results indicate that the size distribution of the W/O/W can be used as a relative proxy for the final size of the antibubbles.

**Fig. 8:**
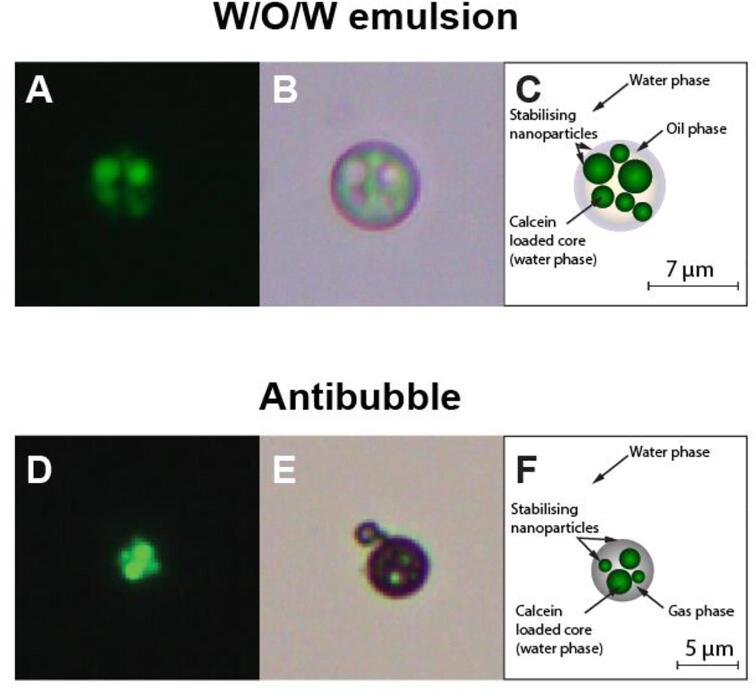
Examples of W/O/W emulsion and resultant PFC antibubble of formulation F2. **Panels A and D** show the fluorescence image visualising the calcein loaded cores. **Panels B and E** show the brightfield image. **Panels C and F** show a schematic illustration of the imaged particle and antibubble. The scale bars are also equivalent the emulsion particle and antibubble diameter.

A comparison of some brightfield and fluorescence microscopy of the W/O/W emulsion and resulting antibubble can be seen in Fig. 8. The fluorescence imaging clearly shows that a minimum of four cores (Fig. 8**D**) are within the antibubble.

**Fig. 9:**
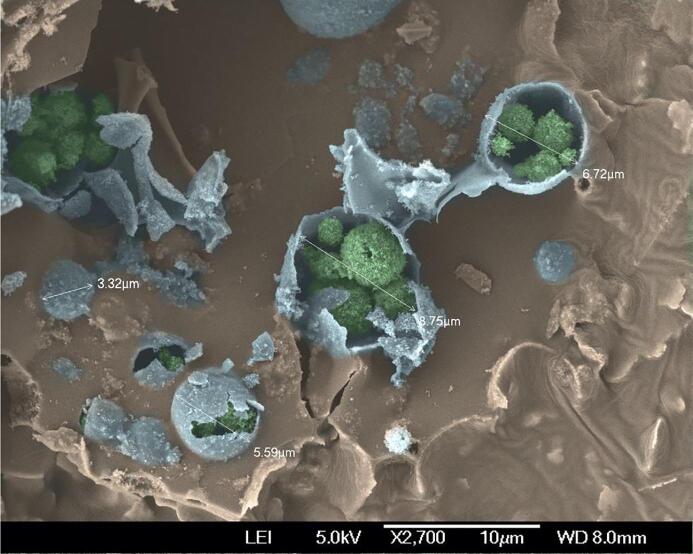
Colourised SEM image of the dry material obtained after lyophilizing the optimized double emulsion formulation F2. The outer silica shell of the antibubbles is shown in blue, and the dried inner droplets of the former W/O/W emulsion are shown in green. The diameter of the individual antibubbles is indicated in the image.

A colourised SEM image of the dry material obtained after lyophilizing the optimized double emulsion formulation F2 can be seen in Fig. 9. Before imaging, a force was applied to the sample in order to deliberately fracture the outer silica shell of the antibubbles. The resulting image shows several bubbles (with a cracked outer silica shell) with a size smaller than 10 µm. These bubbles are filled with multiple spheres. These spheres are the dried inner droplets of the former W/O/W emulsion. Resuspension of the lyophilized material gives a suspension of antibubbles and match those observed in Fig. 8.

One of the envisaged advantages of antibubbles over previously presented drug-loaded bubbles is a higher drug loading. In our work here, the mean volume of an antibubble was 15.2 ± 5.3 (µm)^3^ whilst the mean total core volume for a single antibubble was 3.0 ± 0.4 (µm)^3^ (*i.e.*, 3.0 × 10^-9^ µL). This results in a mean percentage volume loading of 21 ± 6%.

Lentacker et al. coated bubbles with doxorubicin-loaded liposomes and achieved a loading of 3.25 × 10^-8^ μg per bubble [34]. In comparison, from our measurements the average loading of our antibubbles is 3.0 × 10^-6^ µg (assuming water density); a 92-fold increase in drug loading clearly depicting the benefits of antibubbles for targeted drug release. However, the loading capacity is expected to be lower for very lipophilic drugs. Due to low water solubility these may have to be emulsified into the inner water phase, resulting a triple emulsion *i.e.,* as the hydrophobic agent would need to be emulsified into a hydrophilic carrier. Nevertheless, this can be achieved with high yields using modern emulsification techniques. Furthermore, with improved formulation methods, it is expected that an even larger volume can be encapsuled within each antibubble. Previously our simulations showed that increasing the volume of the inner cores would increase the volumetric oscillation amplitude under ultrasonic excitation [35], potentially improving the efficacy of ultrasound mediated drug delivery. This in contrast with other outer shell loading method where an increased drug load is expected to inhibit volumetric oscillations [15].

### 3.2 *In vitro* stability and response of antibubbles to ultrasound

Lyophilized samples containing either air or PFC were examined. Lyophilized samples were either diluted in 0.9% saline or, to better mimic the organic and inorganic components in blood, DMEM medium [36]. The average antibubble size and the concentration for the different conditions are shown in Table 4 (*n* = 3 vials, 5 – 12 images per sample, 977 – 1495 antibubbles per condition). Interestingly, knowing that the volume-averaged size of the bubbles is 5.75 µm, we calculated that the bubble density should be around 10^9^ particles/mL. This is close to the measured values indicating that not many bubbles have been lost. The most important difference between the antibubbles filled with air and antibubbles filled with PFC was observed using brightfield microscopy (Supplemental Fig. 5). For the PFC-filled antibubbles we observe antibubbles smaller than 10 µm that are rather spherical, whereas for the air-filled antibubbles several larger antibubbles with a non-spherical structure are seen. It has previously been shown that bubbles will take up gas from the surrounding fluid and afterwards start to dissolve [37]. As described previously, this may be the phenomenon being observed here, where the antibubbles take up dissolved fluid gasses, *e.g.*, CO_2_, allowing the shell to crack and subsequently the bubble deforms as the air is dissolved into the surrounding fluid. For the PFC antibubbles, due to the hydrophobic nature of the PFC, it does not readily dissolve into the surrounding fluid. Commercial ultrasound-contrast bubbles are filled with hydrophobic high-density gasses (*e.g.*, SF_6_ or C_4_F_10_) as they have extremely low water solubility which increases the stability of the bubbles against dissolution and disproportioning.

**Table 4:** Impact of various gas/media combinations on the antibubbles’ normalised mean size and concentration. A loss in concentration is observed after diluting in DMEM and air filled antibubbles were the least stable.

Gas	Media	Mean size (µm)	Equivalent concentration (x10^8^ particles/mL)
PFC	Saline	3.3 ± 2.5	5.6 ± 1.3
PFC	DMEM	2.9 ± 2.1	5.0 ± 0.5
Air	DMEM	3.0 ± 1.8	3.8 ± 0.7

### 3.3 Attenuation and cavitation response of antibubbles

**Fig. 10:**
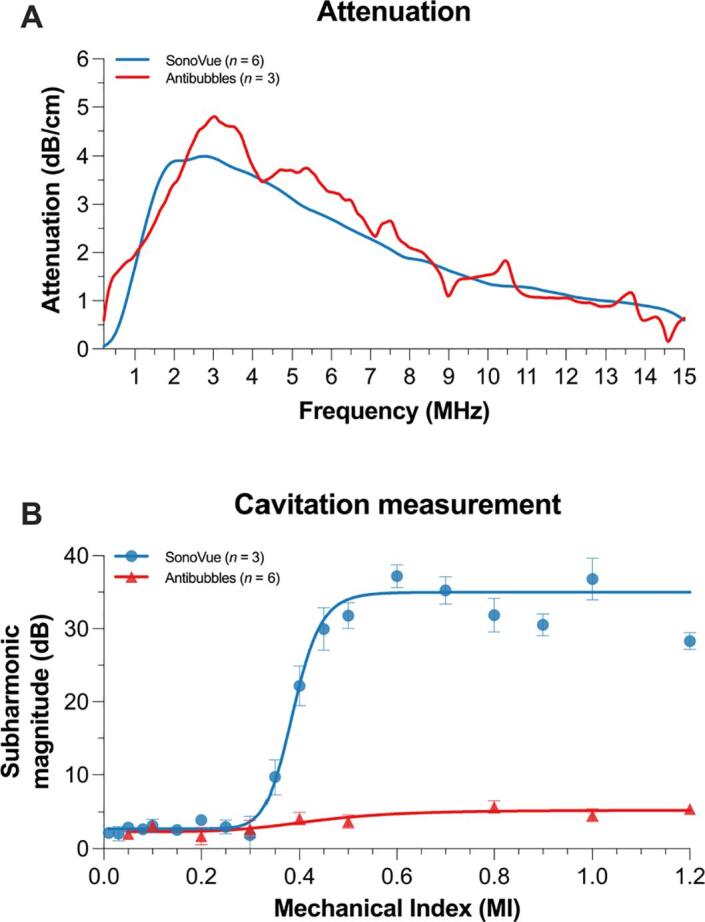
Average attenuation spectra and inertial cavitation measurements. **Panel A**: The antibubbles had a peak attenuation of 4.8 dB/cm at 3.0 MHz; 0.8 dB/cm higher than SonoVue®. **Panel B**: The cavitation measurements showed that the antibubbles did not exhibit any subharmonic signature indicating a different acoustic behaviour than SonoVue®. *n* indicates the number of independent vials, 3 samples were measured from each vial, *i.e.*, 135 – 270 samples per datapoint.

When measuring the acoustic attenuation of antibubbles (n = 3), at a concentration of 200 × 10^3^ particles/mL, a peak attenuation of 4.8 dB/cm was observed at 3.0 MHz (Fig. 10**A**). In comparison, SonoVue® (n = 6), at the same concentration had an attenuation of 4.0 dB/cm at 2.77 MHz. This increase in attenuation may be because the volume occupancy of a similar concentration of antibubbles is larger than that for SonoVue®. A key difference between the antibubbles and SonoVue® was the shape of the curve. Whilst SonoVue® shows a smooth change in attenuation as a function of frequency, the antibubbles have attenuation peaks at 5.5 MHz, 7.5 MHz, 10.5 MHz, and 13.5 MHz, which are not present in SonoVue®. These additional peaks may be due to the different attention induced by antibubbles and non-loaded antibubbles (microbubbles) or may also be due to multiple oscillation modes of antibubbles matching what has previously been simulated [35]. As the antibubbles often have an initial shape that partially deviates from a perfect sphere, the resulting volume oscillations will then tend to be non-spherical. We hypothesise that this will facilitate shape oscillations compared to perfectly spherical microbubbles. In addition, as antibubbles have two low–high density boundaries, this should give more room for surface instabilities which may contribute to a transition into shape oscillations as lower oscillation amplitudes. The non-spherical shape oscillations may contribute significantly to attenuation via absorption but may be ineffective in scattering either due to rapid decay in the far-field or because the divergence of the resulting displacement and the velocity fields are zero. The non-spherical shape oscillations can then potentially drain energy from the antibubble and suppress nonlinear volume oscillations.

The relatively high pressure (0.56 MPa) that was used to excite the antibubbles may be above the critical value for antibubbles (*e.g.,* 0.26 MPa for a commercially available microbubble formulation [38]). Hence, the observed non-linear response at higher frequencies may also be due a lower critical pressure for antibubbles versus SonoVue®. Whilst the impact of acoustic excitation pressure on attenuation has been thoroughly investigated for lipid microbubbles [39] no experimental validation has been performed for antibubbles. To fully elucidate the response of antibubbles to ultrasound excitation, the impact of various acoustic excitation regimes should also be evaluated.

Figure 10**B** shows the mean subharmonic magnitude detected by the broad-band PVDF transducer at 400–600 kHz as a function of MI for both SonoVue® (n = 3) and antibubbles (n = 6). It is assumed as the MI increases, more microbubbles will undergo inertial cavitation that can be detected as an increase in subharmonic magnitude [40]. SonoVue® presented the expected response where at an MI of 0.3 the subharmonic magnitude rapidly increased until it reached its maximum amplitude at an MI of 0.5. The subharmonic magnitude shows a progressive decrease as the MI is increased past the cavitation threshold of 0.5, as microbubbles undergo inertial cavitation in the ultrasound transducer nearfield attenuating the ultrasound signal at the point of measurement. In contrast to SonoVue®, the antibubbles did not display the same behaviour, no increase in subharmonic signal was observed for all the measured MIs (up to MI = 3.0, data not shown). Nevertheless, antibubble destruction could be clearly visualised in the high-speed imaging and flow-phantom ultrasound imaging measurements (see subsequent sections). This indicates that the antibubbles may not undergo the traditional inertial cavitation collapse as other microbubbles but rather a rapid form of stable cavitation that results in antibubble dissolution. A reason for the lack of subharmonic emission could be the asymmetric and non-spherical oscillations of the antibubbles. The asymmetric oscillations may result in more broadband emissions at higher frequencies, nevertheless, evaluating the acoustic spectrum for higher harmonics or broadband noise resulted an identical trend (data not shown). A reason for the inability to detect high frequency broadband emissions may be due to the increased attenuation of high-frequency ultrasound or the sensitivity of the PCD at the higher frequencies. We also hypothesise that these asymmetric oscillations may result in interactions between the core and outer shell of the antibubble further damping the volumetric oscillations, preventing rapid collapse and the generation of subharmonic shockwaves. Furthermore, the non-spherical oscillations may allow for the increased probability of antibubble dissolution by release of shell material forming smaller microbubbles, which may not produce a subharmonic response.

### 3.4 High-speed imaging of antibubbles under ultrasound excitation

**Fig. 11:**
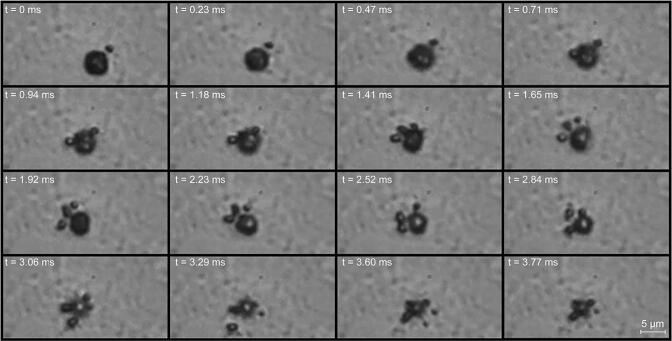
High speed optical microscopy at 127 500 fps of a 5.1 µm diameter antibubble in contact with cells in DMEM at an MI = 0.6. The antibubbles can be visualised to volumetrically oscillate with every ultrasound pulse and shed smaller microbubbles (*c.f.,* Supplemental Video 2). Image size 128 × 32 pixels.

The *in vitro* response of the PCF-filled microbubbles in contact with BxPC-3 (pancreatic cancer) cells in DMEM solution at 25 °C was visualised using high-speed optical imaging. Fig. 11 **and** Supplemental Video 2 shows an example response recorded at 127 500 fps and shutter speed of 1.05 µs. Ultrasound was applied at: MI = 0.6, 0.98 MHz, 60 cycle pulse with one pulse every 100 µs resulting in a calculated spatial peak temporal average intensity (I_SPTA_) of 6.6 W/cm^2^ and spatial peak pulse average intensity (I_SPPA_) of 10.9 W/cm^2^. The antibubble was observed to volumetrically oscillate asymmetrically during ultrasound exposure as assumed during the attenuation measurements. This can be observed in the video where when the ultrasound is applied, the antibubble boundary appears more diffuse or “blurry”. This is due to the antibubble expanding and contracting within each frame exposure period. Specifically, at a shutter speed of 1.05 µs, the antibubble is expected both expand and contract one time.

Over a period of 3.77 ms the antibubbles can be seen shedding small bubbles which also oscillate and stay in close contact with the antibubble, potentially due to secondary Bjerknes forces of acoustic streaming. In parallel to this, the appearance of the antibubble changes; a bright single spot appears in its center. This is an indication of the loss of cores from the antibubbles since this leads to less refraction and scattering of light and the antibubble then appears more similar to traditional microbubbles.

At higher MIs (Supplemental Video 3**,** MI 1.5, 0.98 MHz, 10 cycle pulse with 100 µs resulting in a calculated I_SPTA_ of 3.1 W/cm^2^ and I_SPPA_ of 33.7 W/cm^2^, recorded at 30 000 fps and a shutter speed of 1.05 µs) a similar response was observed albeit significantly faster; *i.e.,* within 0.2 ms the antibubbles core appeared to be released whilst the antibubbles sheds smaller microbubbles and all bubbles dissolve within 20 ms of ultrasound exposure.

Interestingly, the antibubbles are not seen to first grow and then violently collapse, *i.e.* the resulting disintegration does not seem to be the result of inertial cavitation but seems to be the result of stable cavitation and dissolution agreeing with the subharmonic echo measurements. This would be advantageous as it would represent a relatively mild way of drug release with a lower risk of damaging healthy tissue. Nevertheless, this would need to be confirmed using imaging at higher framerates.

Whilst the I_SPTA_ is higher than allowed during diagnostic imaging of 0.72 W/cm^2^ according to IEC 60601–2-37, much higher acoustic intensities are already used for therapeutic ultrasound. As the result show in Supplemental Video 2 occurs within 3.77 ms, allowing for a brief pause in acoustic emission after this period, as is typical in diagnostic ultrasound image formation, to an equivalent ultrasound imaging frame rate of 30 fps would result in an I_SPTA_ of 710 mW/cm^2^. Similarly, for the results in Supplemental Video 2, adding a brief pause after the 20 ms of ultrasound exposure to an equivalent ultrasound imaging frame rate of 10 fps would results in an I_SPTA_ of 604 mW/cm^2^. These values are within the diagnostic threshold, and it would still be expected to release the incorporated drug load. Nevertheless, the antibubble response *in vivo* may be different than that seen *in vitro* meaning higher acoustic intensities may be needed or adjusting other acoustic parameters such as the frequency, bandwidth, pulse length, nonlinearity, and PRF may still result in drug release in an acoustic parameter space compliant with the regulations in diagnostic ultrasound.

### 3.5 *In vitro* ultrasound imaging of antibubbles

When visualising the antibubbles in a flow phantom they could be detected and became more and more visible as the MI was increased (MI 0.05 – 1.2) albeit with an apparent decrease in concentration (Supplemental Video 4). The results indicate that the produced antibubbles act as ultrasound contrast agents and can be readily visualised with clinically utilised ultrasound systems. Previously, for antibubbles >20 µm in diameter, in which the cores were solid, it was also found that clear ultrasound scattering was observed at an MI of 0.1 [41]. The apparent decrease in antibubble concentration at higher MIs can be attributed to antibubble destruction.

Antibubble destruction at an MI of 0.6 can be better visualised in Supplemental Video 5 where no fluid flow is applied. Here, within 60 frames (∼1s) near all antibubbles are visualised to be destroyed. In a flow condition, a similar response is seen (Supplemental Video 6). As the antibubbles flow through the ultrasound imaging field, less bubbles are seen the longer they are imaged. Supplemental Fig. 6**A** shows a frame from the flow condition where the antibubbles are imaged with an MI of 0.6. The antibubbles are flowing from left to right in the image. On the left-hand side, a bright image can be seen within the vessel indicating a high antibubble density. After 1.5–2.0 cm of ultrasound exposure a large decrease in bubble density is observed. By quantifying the image brightness **(**Supplemental Fig. 6**B)** at the top, middle and bottom of the vessel we observe a decrease in image brightness at all three levels after 2.0 cm of ultrasound exposure. This decrease in brightness may be due to bubble destruction or potentially to acoustic radiation force pushing the antibubbles out of the ultrasound imaging field of view or slice. Nevertheless, the impact of acoustic radiation can be better seen in Supplemental Videos 5 & 6**,** allowing a clearer distinction between antibubble destruction and radiation. The use of acoustic radiation to push the antibubbles close to a vessel wall before releasing the drug and inducing “sonoporation” could be advantageous as the proximity to the vessel would be needed to maximise the therapeutic impact.

### 3.6 *In vivo* antibubble stability

**Fig. 12:**
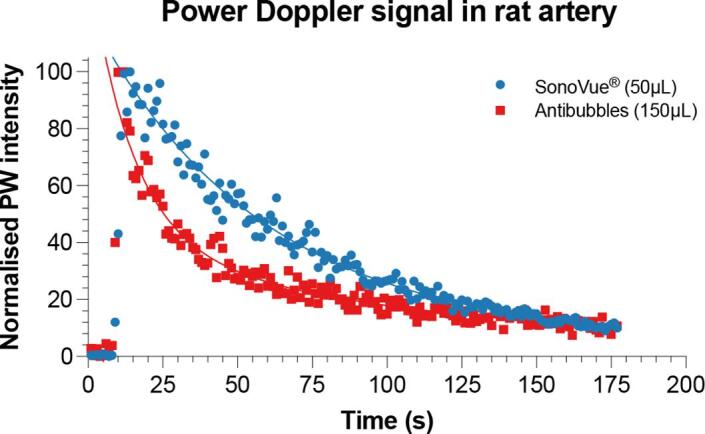
Normalised pulse-wave Doppler signal intensity after intravenous injection of SonoVue and antibubbles in a rat. The solid line indicated the best fit exponential decal model. SonoVue had a one-phase decay (p = 0.134) whilst the antibubbles followed a two-phase decay (p < 0.0001).

Figure 12 compares the normalised pulsed-wave Doppler image intensity of antibubbles *vs* SonoVue® after injection into an anaesthetised rat. Different volumes of antibubbles *vs.* SonoVue® were injected to match the total number of injected bubbles (∼12 × 10^6^ bubbles/antibubbles). A comparison of fits using a nonlinear exponential decay curve determined that SonoVue® had a one-phase decay whilst the antibubbles followed a two-phase decay (p < 0.0001). Using the appropriate models, SonoVue® had a half-life of 40.02 s (R^2^ = 0.98) whilst the antibubbles had a half-life of 9.91 s and 68.49 s for each phase (R^2^ = 0.95). Even though the longer half-life of the antibubbles was 28 s longer than for SonoVue®, the overall signal intensity decreased faster for the antibubbles than SonoVue® indicating that the antibubble portion with the shorter half-life was more dominant acoustically. Each phase of the antibubbles may correlate to the different size of antibubbles, or the antibubbles with and without cores as they are expected to have different acoustic responses. These results indicate that there may be potential to develop an acoustic method for determining if the antibubbles have released their cores and become bubbles, which may allow for a theranostic approach to ultrasound and microbubble mediated drug delivery.

Whilst pickering-stabilized bubbles are known to have an almost infinite lifetime when properly stabilized [42] they have been shown to be sensitive to the presence of surface-active molecules [43]. The significant amount of proteins present in blood may therefore have diminished the lifetime of the antibubbles. Also, the signal intensity of the antibubble suspension was lower than that of the SonoVue® suspension, which may be due to the stiffer shell of the antibubbles or the non-spherical oscillations of the antibubbles resulting in higher harmonics which were not detectable by the ultrasound transducer.

### 3.7 *In vivo* antibubble imaging

**Fig. 13:**
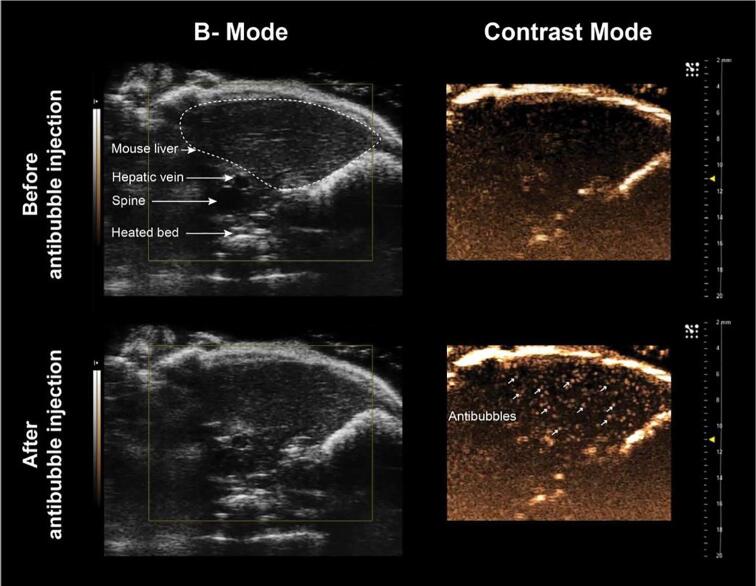
Mouse liver visualised using high-frequency ultrasound before and after (1 m 17 s) antibubble injection. Antibubbles are clearly visualised as bright moving or stable dots in the liver as indicated with the white arrows.

Antibubbles (50 µL) were injected into the lateral tail vein of mice whilst the liver was imaged using high frequency ultrasound. Supplemental Video 7 shows a mouse liver imaged using 18 MHz ultrasound on a Vevo2100 small animal imaging system with contrast mode. The hepatic vein and other key structures are shown in the B-mode image (Fig. 13**A**). Prior to injection of the antibubbles, the mouse liver appears as a mostly homogenous dark structure in the contrast mode (Fig. 13**A** right panel). Antibubbles are visualised approximately 10 s after injection and appear as bright randomly moving dots where each dot is assumed to be a single antibubble. They can be visualised in the right panel which used non-linear contrast imaging whereas almost no change in signal is observed in the B-mode image indicating the antibubbles are highly non-linear. They remained visible for at least 100 s after injection, confirming the applicability of antibubbles as contrast agents. A single frame 1 m 17 s after antibubble injection is shown in Fig. 13**B**. Whilst almost no difference is seen in the B-mode image, numerous dots can be seen in the liver in the contrast image, *i.e.,* antibubbles in blood vessels and capillaries. The wite arrows point to 10 randomly selected examples of the hundreds of dots (antibubbles) that can be visualised. Some of the antibubbles are also observed as static dots, akin to that seen in acoustic cluster therapy [44], indicating they may have lodged or fused with the vessel wall. The antibubbles may coalesce to form larger single bubbles upon ultrasound application. The amount of static antibubbles increased with time.

When imaging using a clinical ultrasound system (GE Logiq E9, Supplemental Video 8) a similar result was observed. The hepatic vein can be seen in the lower left quadrant and the antibubbles initially appear at approximately 10 s after injection. The antibubbles can be clearly seen in the non-linear imaging mode (right panel) as bright dots that flow throughout the vasculature but almost no change is observed in the B-mode image. Yet again a few static dots appear, but they are more difficult to distinguish due to the lower image resolution.

No acute toxicity was observed following injection of antibubbles nor any other adverse effects manifesting as behavioural changes indicating pain during the follow-up period. Further toxicology studies should be performed to evaluate organ specific nanoparticle uptake and excretion mechanisms and other potential organ specific damage.

### 3.8 *In vitro* model drug delivery

**Fig. 14:**
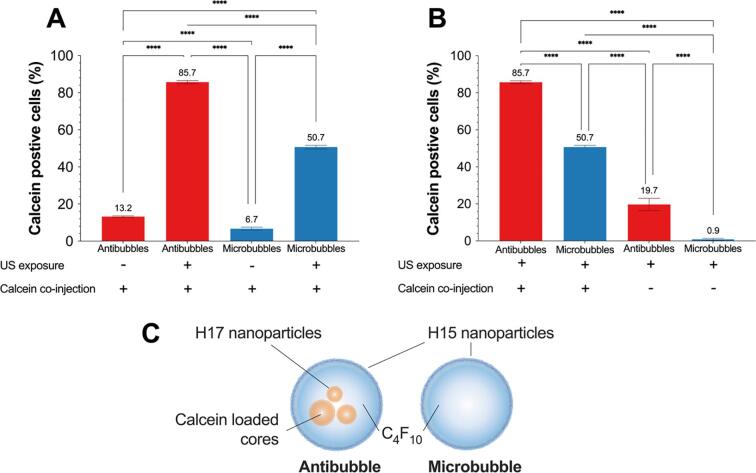
Sonoporation efficacy of antibubbles compared to nanoparticle stabilised microbubbles. **Panel A** compares the impact of ultrasound when performing sonoporation with the model drug co-administered. **Panel B** compares the impact of co-administering the model drug. **Panel C** is a graphical representation of an antibubble and microbubble. In all directly comparable results, the antibubble delivered model drug to significantly more cells than the microbubbles. *n* = 3 for each sample.

To evaluate the potential of the antibubbles to enhance uptake of a model drug, sonoporation was induced using calcein as the model drug. To separate the impact of the non-loaded antibubbles (nanoparticle stabilised microbubbles), a control group of nanoparticle stabilised microbubbles was also produced (Fig. 14**C**). The results show that when using ultrasound and antibubbles with a calcein core whilst co-injecting calcein into the solution, the antibubbles were able to deliver calcein to 85.7% of cells, whilst the nanoparticle stabilised microbubbles at the same concentration were able to deliver co-injected calcein to 50.7% of the cells, a 69% increase in efficacy (p < 0.0001) (Fig. 14**A**). Applying ultrasound to the antibubbles increased the uptake from 13.2% to 85.7% indicating that the enhanced uptake was due to sonoporation rather than endocytosis. Calcein was co-injected also for the antibubble samples to match the calcein concentration in solution. Specifically, the injected volume of calcein in the microbubble sample was 3 µL whilst the volume of calcein within the antibubbles was calculated to be approximately 0.006 µL, *i.e.*, 500 × less. For the given experimental configuration, reducing the injected concentration was previously shown to be unreliable.

When no calcein was co-injected the microbubbles resulted in 0.9% calcein positive cells (due to autofluorescence) whereas the antibubbles resulted in 19.7% calcein positive cells, indicating that the antibubbles can indeed release their core and deliver it to the cells (Fig. 14**B**). This also indicates that the majority of calcein delivered to the cells in the calcein co-injection experiments is from the co-injected calcein.

A higher concentration of antibubbles may result in more antibubble-cell contact and a higher percentage of calcein positive cells.

### 3.9 Limitations and future work

Whilst this initial work on micron-sized antibubbles shows promise as a novel method of ultrasound guided drug delivery, it is only a proof of concept on the potential for antibubbles. A major limitation with the current formulation of the antibubbles is that less than approximately 40% of the produced formulation are truly antibubbles (*i.e.*, with cores). As a consequence, it may be difficult to differentiate the impact of nanoparticle stabilised microbubbles *vs*. antibubbles during ultrasound imaging and sonoporation treatment. Hence to better separate the impact and behaviour of antibubbles, a formulation technique that yield a higher proportion of antibubbles is needed. An alternative is to explore techniques such as buoyancy-based filtration followed by a re-concentration method.

In addition, the current formulation uses silica nanoparticles which may have unknown systemic toxicities when used for intravascular injection. Whilst the toxicity of silica nanoparticles after intravascular administration has been evaluated previously [45], [46] a more in-depth toxicology study would be needed, alternatively exploring the use of more established hydrophobic nanoparticles such as PLGA which are approved by the US FDA as a drug delivery system [47].

In therapeutic ultrasound, a long microbubble *in vivo* half-life is expected to be most effective for enhancing therapy as a longer half-life allows the microbubbles to circulate multiple times across the target site, increasing the probability of every microbubble inducing a therapeutic action. This is also of great importance for drug loaded microbubbles as this ensures minimal drug is releases at non-target sites. This formulation has a marginally better half-life than conventional diagnostic microbubbles, which may be suboptimal. Nevertheless, previous research has shown that in solution, antibubbles can be stable for significantly longer, hence further formulation optimisations may improve the *in vivo* stability. Nonetheless, the key challenge is balancing between a more solid shell for increased *in vivo* stability and a soft, more gas permeable, shell for better therapeutic efficacy and drug release at low ultrasound intensities. It should also be considered that many oncological diseases are considered systemic diseases, hence both systemic and enhanced local release and delivery might have further real-world advantages over purely local release and delivery.

Whilst the current formulation shows improvements in drug loading compared to previous drug-loaded microbubble concepts, further improvements need to be made to increase the drug volume and concentration. In addition, the use of lipophilic drugs rather than model drugs needs to be evaluated.

## 4 Conclusions

In this work we have shown that model drug-loaded antibubbles can be produced with a mean size of 2.96 ± 1.94 μm with 90% below 5.5 µm, and 99% below the size limit of <10 μm as needed to pass through blood capillaries. These antibubbles were also shown to be able to release their model drug core upon exposure to clinical ultrasound settings under *in vitro* conditions. The antibubbles were detectable and showed similar stability to commercial ultrasound contrast agents *in vitro* and *in vivo,* and may therefore function as contrast agents for imaging. Furthermore, *in vivo* experiments showed no acute toxicity after injection of the antibubbles into mice or rats. In addition, the antibubbles are produced by freeze-drying which allows long term storage and transport. More research is needed to demonstrate ultrasound-triggered release and efficacy under *in vivo* conditions and to better determine the safety of the antibubbles. However, based on the results so far, we believe that antibubbles represent a promising new approach for ultrasound-triggered drug delivery with a potentially higher drug loading and better stability of the loaded drug than current drug-loaded bubbles.

## Funding

This study was funded by the Western Health Board of Norway (Grant numbers 911779, 911182, 912035 and 912146), by the Norwegian Cancer Society (6833652, 182735) and by the Norwegian Research Council (SonoCURE grant no. 250317), Research Council of Norway through its Centers of excellence funding scheme, project numbers 223250 and 262652.

### CRediT authorship contribution statement

**Spiros Kotopoulis:** Conceptualization, Data curation, Formal analysis, Funding acquisition, Investigation, Methodology, Project administration, Resources, Supervision, Validation, Writing – original draft, Writing – review & editing. **Christina Lam:** Data curation, Investigation, Methodology, Writing – original draft, Writing – review & editing. **Ragnhild Haugse:** Data curation, Investigation, Methodology, Project administration, Resources, Supervision, Writing – review & editing. **Sofie Snipstad:** Data curation, Investigation, Methodology, Resources, Writing – review & editing. **Elisa Murvold:** Investigation, Methodology, Writing – review & editing. **Tæraneh Jouleh:** Writing – review & editing. **Sigrid Berg:** Resources, Writing – review & editing. **Rune Hansen:** Investigation, Methodology, Project administration, Resources, Writing – review & editing. **Mihaela Popa:** Investigation, Methodology, Writing – review & editing. **Emmet Mc Cormack:** Funding acquisition, Resources, Supervision, Writing – review & editing. **Odd Helge Gilja:** Funding acquisition, Resources, Supervision, Writing – review & editing. **Albert Poortinga:** Conceptualization, Investigation, Methodology, Resources, Writing – original draft, Writing – review & editing.

## Declaration of Competing Interest

The authors declare that they have no known competing financial interests or personal relationships that could have appeared to influence the work reported in this paper.
